# Whole-Transcriptome Profiling and circRNA-miRNA-mRNA Regulatory Networks in B-Cell Development

**DOI:** 10.3389/fimmu.2022.812924

**Published:** 2022-03-21

**Authors:** Jie Pan, Saineng Hu, Xuanyao Ren, Hao Hu, Xiaoying Deng, Bo Yu, Inma Cobos, Xiaofan Chen, Wei Zhang

**Affiliations:** ^1^ Biomedical Research Institute, Shenzhen Peking University - The Hong Kong University of Science and Technology Medical Center, Shenzhen, China; ^2^ Department of Pathology, Stanford University School of Medicine, Palo Alto, CA, United States; ^3^ Department of Dermatology, Peking University Shenzhen Hospital, Shenzhen, China; ^4^ Greater Bay Biomedical Innocenter, Shenzhen Bay Laboratory, Shenzhen, China

**Keywords:** circRNA, ceRNA network, B-lymphocyte development, B-lymphocyte differentiation, RNA-seq

## Abstract

The generation and differentiation of B lymphocytes (B cells) is a flexible process with many critical regulatory factors. Previous studies indicated that non-coding RNAs play multiple roles in the development of lymphocytes. However, little has been known about the circular RNA (circRNA) profiles and their competing endogenous RNA (ceRNA) networks in B-cell development and differentiation. Here, four B-cell subsets were purified from single-cell suspensions of mouse bone marrow. Then RNA sequencing (RNA-Seq) was used to display expression profiles of circRNAs, miRNAs and mRNAs during B-cell differentiation. 175, 203, 219 and 207 circRNAs were specifically expressed in pro-B cells, pre-B cells, immature B cells and mature B cells, respectively. The circRNA-associated ceRNA networks constructed in two sequential stages of B-cell differentiation revealed the potential mechanism of circRNAs in these processes. This study is the first to explore circRNA profiles and circRNA-miRNA-mRNA networks in different B-cell developmental stages of mouse bone marrow, which contribute to further research on their mechanism in B-cell development and differentiation.

## Introduction

**Figure 1:**
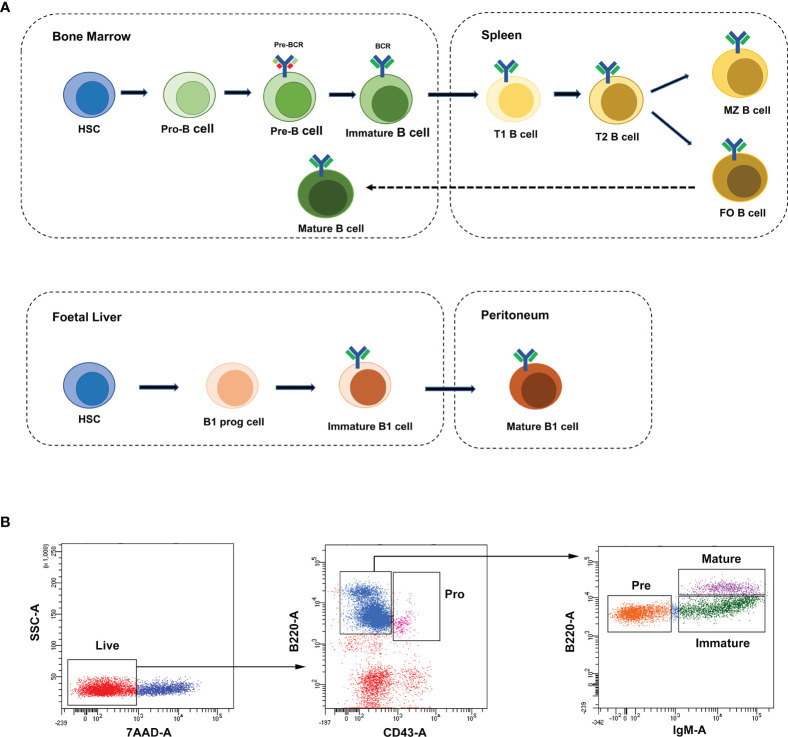
B-cell development stages and cell sorting strategies. **(A)** The broad outline of B-cell development stages in mice and humans. Solid arrows indicate the progression of B-cell development. Dashed line indicates recirculation of follicular B cells back to the bone marrow. **(B)** The FACS sorting strategies of B cells in mouse bone marrow. These cell populations were defined as pro-B cells (Pro, B220^+^IgM^-^CD43^+^), pre-B cells (Pre, B220^+^IgM^-^CD43^-^), immature B cells (Immature, B220^+^IgM^+^CD43^-^) and mature B cells (Mature, B220^hi^IgM^+^CD43^-^).

B lymphocytes (B cells) were defined as a group of lymphocytes that express clonally diverse cell-surface immunoglobulin receptors (1), which were discovered in the 1960s (2, 3). Mouse and human lymphocytes are generated from pluripotent hematopoietic stem cells (HSCs) in the fetal liver and adult bone marrow (BM) (4). Nevertheless, B cells develop in the bone marrow, while T lymphocytes (T cells) are formed in the thymus. The B cell receptor (BCR) is a membrane immunoglobulin (mIg), which is essential for B cell development and survival (5). Under the recombinase activity of Rag1/Rag2 (recombination activating gene, RAG) endonuclease, the BCR was formed through the rearrangements of both V, D, J gene segments (in the H chain locus) and V, J gene segments (in the L chain locus) in the Ig gene (6, 7). As shown in **Figure 1A**, according to the rearrangement of Ig genes and expression of cell surface markers, the development of B cells in mouse bone marrow can be defined into four stages: pro-B cells, pre-B cells, immature B cells and mature B cells (8). The pro-B cells come from the hematopoietic-cell lineages. After the μ-heavy chain was formed through the rearrangement of V-D-J gene, pre-B-cell receptor (pre-BCR) expressing pre-B cells were derived from pro-B cells. After the light chain was formed through the rearrangement of V-J gene, pre-B cells switched to immature B cells, which expressed membrane-bound IgM (mIgM) of the B cell receptor complex. The immature B cells migrate from the bone marrow to the spleen and then differentiate into mature follicular (FO) B cells or marginal zone (MZ) B cells (8–10).

Non-coding RNAs (ncRNAs) include long non-coding RNAs (lncRNAs), microRNAs (miRNAs) and circular RNAs (circRNAs). The biological functions of these ncRNAs have been recognized in the past decades. MiRNAs are a type of about 22-nucleotide-long and single-stranded RNA molecules, which control various biological processes. MiRNAs could inhibit the translation of the messenger RNAs (mRNAs) through interacting with their 3’-untranslated regions (UTR) (11). Unlike linear non-coding RNAs, circRNAs form covalently closed continuous loop structures without 3’-poly (A) and 5’-cap. CircRNAs were abundant, highly stable and conserved in animals and humans (12). The spatiotemporally specific expression patterns of circRNAs suggest their potential functions in physiological processes and pathobiology (13, 14). An increasing number of studies have revealed that circRNAs play significant roles in carcinogenesis (15), immune disorders (16), cardiovascular diseases (17) and neurological disorders (18). Endogenous circRNAs are involved in gene regulation by affecting the splicing of their linear mRNA counterparts, regulating transcription of their parental genes, interacting with proteins and being translated into polypeptides (13). Besides the above functions, they also act as miRNA sponges and regulate miRNA-targeted gene expression (19). For example, *Cdr1as* (as known as *ciRS-7*) contains over 70 binding sites for miR-7 (19), which may regulate the expression of miR-7-targeted genes in tumors (20, 21) and neuropsychiatric disorders (22). These circRNAs were considered as competitive endogenous RNAs (ceRNAs) for these mRNAs. Based on the ceRNA hypothesis, the circRNA-miRNA-mRNA networks may play crucial roles in biological pathways (23).

In the past few years, the significant roles of lncRNAs and miRNAs in B-lymphocyte development have been elucidated (24, 25). *BCALM*, a B cell-specific lncRNA, regulated B-cell differentiation through modulating BCR-mediated calcium signaling (26). Meanwhile, microRNAs have been identified to be crucial for regulating BCR signaling (27). The miR-29 family had been proven to regulate B-cell terminal differentiation and survival (28), and miR-29c could regulate Rag1 expression and modulate V(D)J recombination during B cell development (29). Besides, miR-191 has been identified to modulate B-cell development *via* targeting transcription factors E2A, Foxp1, and Egr1 (30). However, the roles of circRNAs in B-lymphocyte development have remained unclear.

To gain further insight into the molecular events associated with B-cell development and differentiation, high-throughput sequencing and integrated analysis of whole-transcriptome were used to investigate the characteristic expression of circRNAs, miRNAs and mRNAs during B-cell maturation. Furthermore, circRNA-miRNA-mRNA networks were established to explore their regulatory roles in B-cell development. This study is the first exploration of circRNA profiles and circRNA-miRNA-mRNA networks of B-cell development in mouse bone marrow, which is valuable for future studies of the mechanism of ncRNAs in B-cell development and differentiation.

## Material and Method

### Mice

All animal experiments were performed with female C57BL/6 mice aged 8-10 weeks, which were purchased from Beijing HFK Bioscience CO.LTD. The mice were kept in a specific-pathogen-free (SPF) environment and provided free access to a standard diet until they met age requirements. Each pool of samples was a mixture of cells from three mice. All procedures were approved by the Animal Use and Care Committee of Shenzhen Peking University - The Hong Kong University of Science and Technology Medical Center (SPHMC) (protocol number 2011-004). Efforts were made to minimize suffering and the number of animals used.

### FACS Sorting of B Cell

Single-cell suspensions of mouse bone marrow were prepared in PBS with 2% FBS. The following reagents were used for cell staining: B220-FTIC (Biolegend, 103206), IgM-APC (Biolegend, 406509), CD43-PE-Cy7 (Biolegend, 143209) and 7-AAD (Biolegend, 420404). B-cell stages in bone marrow were defined with the following gating strategies: pro-B cells (B220+IgM-CD43+), pre-B cells (B220+IgM-CD43-), immature B cells (B220+IgM+CD43-) and mature B cells (B220hiIgM+CD43-). In all cases, cells were gated on live cells (negative for dead cell stain, 7-AAD) and were sorted on FACSAriaIII (BD). Data were analyzed with the BD FCS Diva v8.0.1 software shown in **Figure 1B**.

### RNA Sequencing

RNA extraction and qualification, library preparation, sequencing, quality control, read mapping to reference genome and expression analysis was shown in **Supplementary Methods**.

### Quantitative RT-PCR Validation of Selected Genes

Flow-cytometry-sorted B-cells were used for RNA extraction. cDNA was synthesized from total RNA by random primer/miR-specific RT primers using a Reverse Transcription System (Promega). Quantitative RT-PCR was performed in triplicate in 96-well plates using a qPCR machine (LC480, Roche) and SYBR Green I Master mixture (4887352001, Roche) for detection of amplification products. The following thermocycling protocol was used: initial denaturation at 95°C for 10 min, followed by 40 amplification cycles of 95°C for 15 s and 60°C for 1 min, and a final cycle at 25°C for 15 s. Relative quantification of RNA expression was performed using the comparative cycle method to obtain the following ratio: gene of interest/*Gapdh or U6*. Relative quantification of gene expression levels was performed using the 2^-ΔΔCt^ method.

### Analysis and Construction of ceRNA Networks

The circRNAs, miRNAs and mRNAs with differential expression during distinct B-cell developmental stages were further analyzed for ceRNA networks. CircRNAs were blasted against circBase for annotation. Some of them cannot be annotated, which were defined as novel circRNAs. The relationship between miRNAs and circRNAs annotated in circBase can be predicted by Starbase (version 2.0). Three software including TargetScan (version 7.0), miRanda (version 2.0) and miReap were used to predict targets of novel circRNAs for animal samples. Then, miRTarBase (version 6.1) was used to predict mRNAs targeted by miRNAs sponge. Eventually, based on the ceRNA hypothesis and data described above, circRNA-miRNA-mRNA networks were constructed and visually displayed with Cytoscape-software (version 3.5.0).

### KEGG Pathway and GO Annotations Analysis

The ceRNA-associated target mRNAs and the parental genes of stage-specific circRNAs were analyzed to further investigate their biological functions and pathways through Gene Ontology (GO) functional annotation and Kyoto Encyclopedia of Genes and Genomes (KEGG) pathway analysis with the clusterProfiler R package. GO terms and KEGG pathways with corrected p values < 0.05 were considered significantly enriched.

### Graphs and Statistical Analyses

All statistical analyses were performed using GraphPad Prism 8.00 software (GraphPad Software, La Jolla, CA, USA). Two normally distributed groups were compared using t-tests; p < 0.05 was considered statistically significant.

## Results

### Global Expression Profiles of circRNAs, miRNAs and mRNAs in Distinct Developmental Stages of B Cells

**Figure 2:**
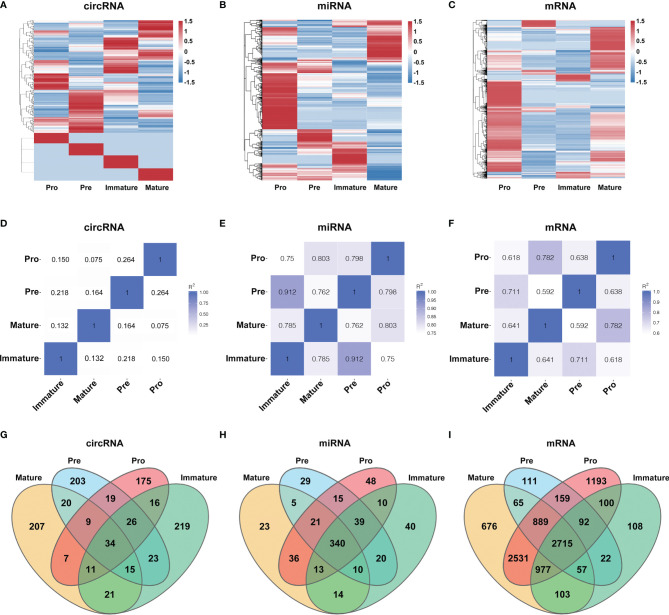
Global gene expression and lineage-specific expression of circRNAs. **(A–C)** The cluster analysis on the expression of the circRNAs **(A)**, miRNAs **(B)** and mRNAs **(C)**. Red and blue: upregulated expression and downregulated expression, respectively. **(D–F)** Heatmap analysis of differentially expressed circRNAs **(D)**, miRNAs **(E)** and mRNAs **(F)** in B-cell subsets. Correlation is evaluated by Pearson’s correlation coefficient of total transcripts expression levels. **(G–I)** Cell-specific expression patterns of circRNAs **(G)**, miRNAs **(H)** and mRNAs **(I)** among distinct B-cell development stages were presented as Venn diagrams; numbers around perimeter indicated the frequency of genes.

To characterize the temporal expression patterns of circRNAs, miRNA and mRNA and their ceRNA networks during the development of B lineages, fluorescence-activated cell sorting (FACS) was used to purify four B-cell lineage populations from the bone marrow of C57BL/6 mice (**Figures 1A, B**). These populations included pro-B cells, pre-B cells, immature B cells and mature B cells (**Figures 1A, B**). Finally, we identified all 1005 circRNAs, 1600 miRNAs, and 9758 mRNAs expressed in at least one of four B-cell subsets. The heatmaps were constructed to show the cluster analysis results of the circRNAs, miRNAs and mRNAs (**Figures 2A–C**). 297, 349, 365, and 324 circRNAs were expressed in the pro-B cells, pre-B cells, immature B cells and mature B cells respectively.

To evaluate the differential expression profiles in different stages, pairwise comparison of circRNAs, miRNAs and mRNAs in any two B-cell development stages was performed with Pearson’s correlation coefficient (**Figures 2D–F**). Contrary to miRNAs and mRNAs, circRNAs were expressed in a stage-specific and lineage-specific manner during B-cell differentiation. We further identified 58%-64% of expressed circRNAs were stage-specific, while only 5%-10% of miRNAs and 2%-14% of mRNAs were stage-specific (**Figures 2G–I**). 175, 203, 219 and 207 circRNAs were specifically expressed in pro-B cells, pre-B cells, immature B-cell, and mature B-cell stages, respectively (details in **Table S1**). In summary, the highly stage-specific expression of the circRNAs indicated their particular functions during B-cell development and differentiation.

### Parental Genes of Stage-Specific circRNAs Were Enriched in the BCR Signaling Pathway

To further investigate the roles of stage-specific circRNAs in B-cell development (details in **Table S1**), the functional enrichment analyses of their parental genes were performed. KEGG pathway analysis revealed biology pathways significantly enriched in different B-cell subpopulations. Phosphatidylinositol signaling system and PD-L1 expression/checkpoint pathway were enriched in pro-B cells. A total of 77 function pathways were enriched in pre-B cells. The top terms included B-cell receptor (BCR) signaling pathway, growth hormone synthesis, secretion and action, chemokine signaling pathway, etc. In the immature B cells, the BCR signaling pathway was the only pathway significantly enriched. As for mature B cells, the T cell receptor signaling pathway, BCR signaling pathway, and MAPK signaling pathway were three top terms in 77 pathways. The top 5 pathways of each B-cell population are listed in **Table 1**, while other pathways can be seen in **Table S2**. The BCR signaling pathway was significantly enriched in multiple B-cell subpopulations, which indicated that circRNAs might be indirectly involved in the BCR pathway by regulating their parental genes.

**Table 1:** The top 5 KEGG pathways of stage-specific circRNAs enriched in four B-cell subpopulations.

Type	Pathway ID	Description	GeneRatio	*P* value	geneID
pro-B cell	mmu04070	Phosphatidylinositol signaling system	7/81	0.00511	Pik3r1/Dgkd/Pip5k1b/ Prkcb/Mtmr3/Inpp5d/ Cds1
	mmu05235	PD-L1 expression and PD-1 checkpoint pathway in cancer	6/81	0.01461	Pik3r1/Traf6/Tlr9/ Map3k3/Stat1/Jak1
pre-B cell	mmu04662	B cell receptor signaling pathway	9/70	2.32E-06	Prkcb/Rac2/Sos2/Pik3r1/ Ppp3ca/Mapk1/Cd22/ Syk/Cd79a
	mmu04935	Growth hormone synthesis, secretion and action	8/70	0.00032	Prkcb/Gnas/Crkl/ Sos2/Pik3r1/Mapk1/ Irs1/Jak2
	mmu04062	Chemokine signaling pathway	9/70	0.00103	Prkcb/Rac2/Crkl/Sos2/ Pik3r1/Mapk1/Elmo1/ Pik3cg/Jak2
	mmu04072	Phospholipase D signaling pathway	8/70	0.00103	Gnas/Dgkz/Sos2/ Pik3r1/Mapk1/Syk/ Pik3cg/Cyth1
	mmu04650	Natural killer cell mediated cytotoxicity	7/70	0.00175	Prkcb/Rac2/Sos2/ Pik3r1/Ppp3ca/ Mapk1/Syk
immature B cell	mmu04662	B cell receptor signaling pathway	6/95	0.04328	Blnk/Syk/Plcg2/ Cd22/Grb2/Prkcb
mature B cell	mmu04660	T cell receptor signaling pathway	10/87	8.61E-06	Nfkb1/Akt2/Tec/Gsk3b/ Ptprc/Nfatc1/Fyn/Nfatc3/ Map2k1/Kras
	mmu04662	B cell receptor signaling pathway	9/87	8.61E-06	Nfkb1/Akt2/Gsk3b/ Inpp5d/Nfatc1/Prkcb/ Nfatc3/Map2k1/Kras
	mmu04010	MAPK signaling pathway	13/87	0.00035	Nfkb1/Rap1b/Akt2/ Rras2/Nfatc1/Prkcb/Fgfr2 /Nfatc3/Mknk2/Map2k1/ Nr4a1/Dusp10/Kras
	mmu04022	cGMP-PKG signaling pathway	10/87	0.00036	Atp2a3/Atp2a2/Akt2/ Irs2/Nfatc1/Prkce/Nfatc/ Slc25a5/Map2k1/Vasp
	mmu01521	EGFR tyrosine kinase inhibitor resistance	7/87	0.00048	Akt2/Gsk3b/Prkcb/Fgfr2/ Map2k1/Kras/Il6ra

### Dynamic Transcriptional Profiles at Distinct B-Cell Developmental Stages

**Figure 3:**
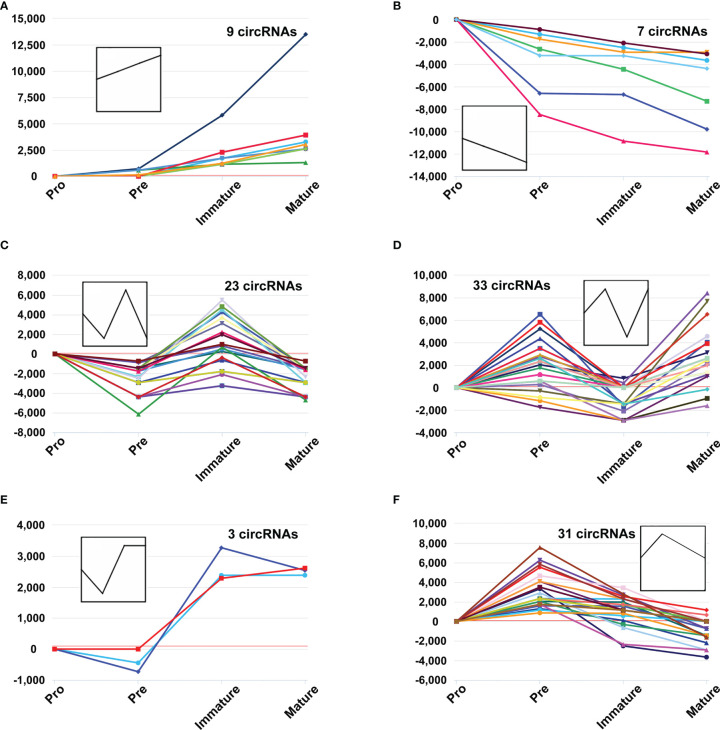
The dynamic expression patterns of CircRNAs during B-cell differentiation. The y-axis represents reads per million (RPM) values at distinct B-cell developmental stages, which were normalized to pro-B cells, while the polylines indicate expression variance trends during stage progressions **(A–F)**.

Several circRNAs expression patterns were identified during B-cell differentiation with the Short Time-series Expression Miner (STEM). Six typical patterns were shown in **Figure 3**, while others were shown in **Supplementary Figure 1** (details in **Table S3**). 9 circRNAs exhibited continuously increased (**Figure 3A**), while 7 circRNAs continuously decreased (**Figure 3B**). On the other hand, other circRNAs showed irregular expression patterns. For example, 23 circRNAs firstly decreased in the pre-B-cell stage, then increased in the immature B-cell stage, and finally decreased in the mature B-cell stage (marked as ‘down-up-down’ pattern) (**Figure 3C**), while 33 circRNAs exhibited ‘up-down-up’ patterns (**Figure 3D**). In addition, the expression of 3 circRNAs slightly decreased in the second stage, then increased and maintained a high level in the latter immature and mature B-cell stages (**Figure 3E**). The expression of 31 circRNAs firstly reached a relatively high level in the second stage, but then continuously decreased in the latter immature and mature B-cell stages (**Figure 3F**). Overall, these data suggested that the expression pattern of circRNAs showed highly dynamic changes during B-cell development.

**Figure 4:**
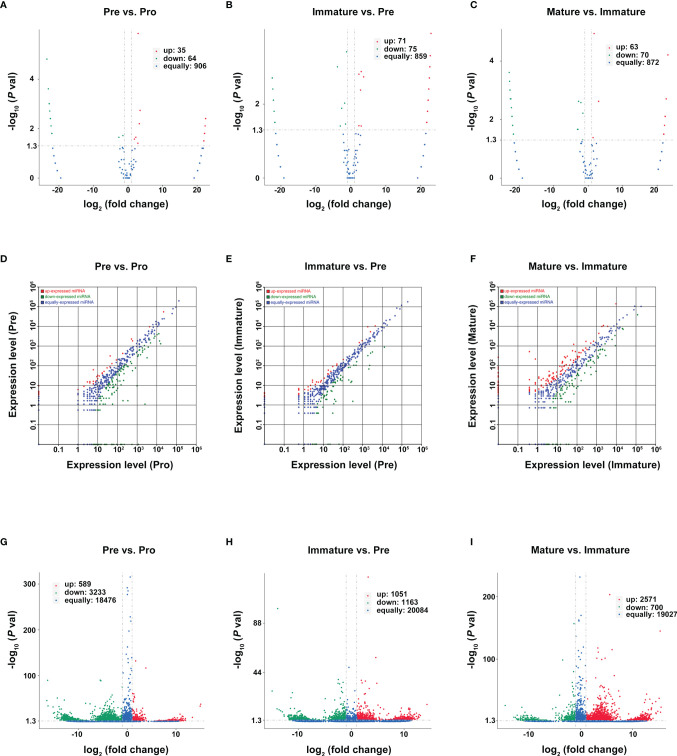
Differentially expressed RNAs during B-cell differentiation stages. **(A–C)** Volcano plot of DEcircRNAs during B-cell differentiation stages. **(A)** Pre vs. Pro, **(B)** Immature vs. Pre, **(C)** Mature vs. Immature. Red and green points represent increased and decreased expression of circRNAs, respectively. X-axis: log2 ratio of circRNA expression levels, y-axis: false-discovery rate values of circRNAs (-log10 transformed). Analysis of DE miRNAs **(D–F)** and DEmRNA **(G–I)** are shown similarly.

Genes with a p-value of <0.05 and log2FC (fold change) ≥ 1 were considered differentially expressed genes between two adjacent developmental stages. Based on reads per million (RPM) values, there were 35 upregulated circRNAs and 64 downregulated circRNAs during the pro-B to pre-B cell transitional stage (marked as Pre vs. Pro group, **Figure 4A**), 71 upregulated circRNAs and 75 downregulated circRNAs during the pre-B to immature B cell transitional stage (marked as Immature vs. Pre group, **Figure 4B**), as well as 63 upregulated circRNAs and 70 downregulated circRNAs during the immature B to mature B cell transitional stage (marked as Mature vs. Immature group **Figure 4C**).

Then, we used the TPM values and the FPKMs value to evaluate the expression levels of miRNAs and mRNAs, respectively. A total of 181 DEmiRNAs (39 upregulated and 142 downregulated) and 3822 DEmRNAs (589 upregulated and 3233 downregulated) were identified during the pro-B to pre-B cell transitional stage (**Figures 4D, G**). 109 DEmiRNAs (39 upregulated and 70 downregulated) and 2214 DEmRNAs (1051 upregulated and 1163 downregulated) were identified during the pre-B to immature B cell transitional stage (**Figures 4E, H**). Moreover, 254 DEmiRNAs (128 upregulated and 126 downregulated) and 3271 DEmRNAs (2571 upregulated and 700 downregulated) were identified during the immature B to mature B cell transitional stage (**Figures 4F, I**). All detailed information is listed in **Tables S4**–**S6**. Consequently, it revealed a highly regulated and dynamic transcriptome during B-cell differentiation.

### Validation of RNA-Seq Profiles by Using qPCR

**Figure 5:**
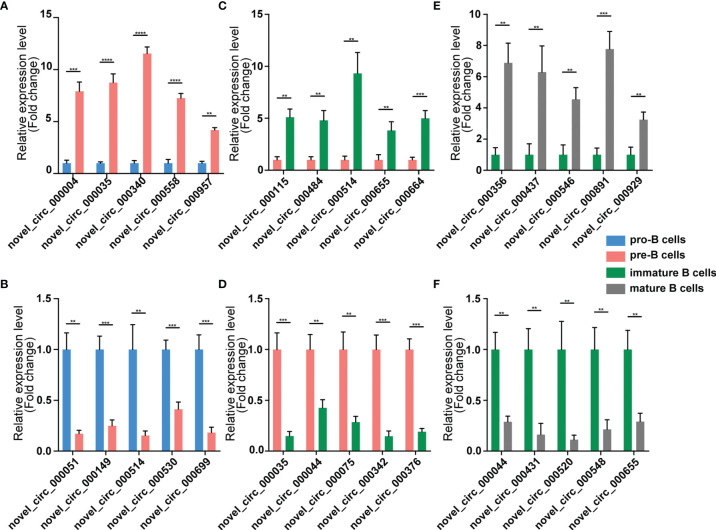
Validation of circRNA expression by using qPCR. The identified differentially expressed transcripts circRNAs were divided into three groups: Pre vs. Pro, Immature vs. Pre and Mature vs. Immature. **(A, B)** Pre vs. Pro, **(C, D)** Immature vs. Pre, **(E, F)** Mature vs. Immature. circRNA expression was quantified relative to the gapdh expression level by using the comparative cycle threshold method. Columns represent means ± SEM; ****p < 0.0001, ***p < 0.001, **p < 0.01.

**Figure 6:**
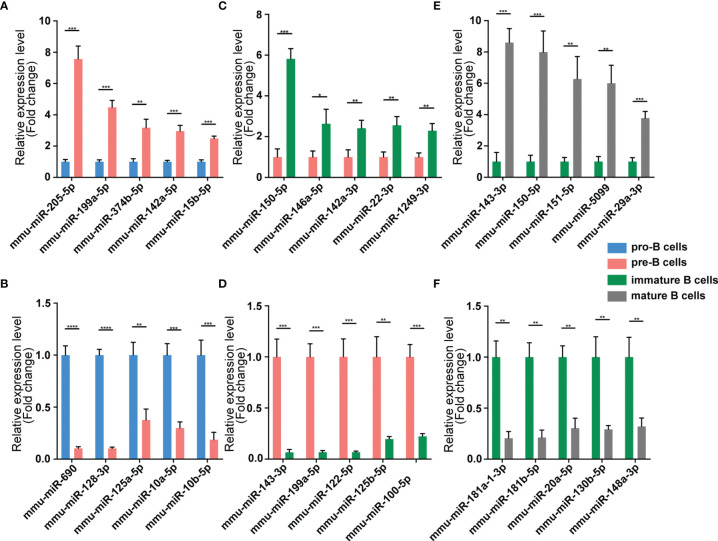
Validation of miRNA expression by using qPCR. The identified differentially expressed transcripts miRNAs were divided into three groups: Pre vs. Pro, Immature vs. Pre and Mature vs. Immature. **(A, B)** Pre vs. Pro, **(C, D)** Immature vs. Pre, **(E, F)** Mature vs. Immature. miRNA expression was quantified relative to the U6 expression level using the comparative cycle threshold method. Columns represent means ± SEM; ****p < 0.0001, ***p < 0.001, **p < 0.01, *p < 0.05.

**Figure 7:**
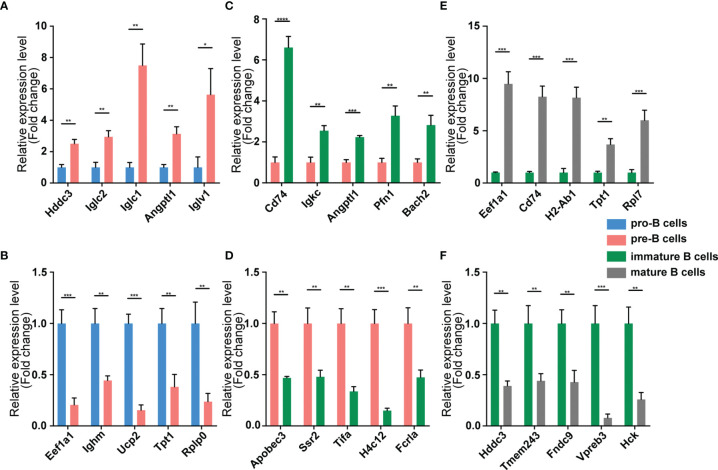
Validation of mRNA expression by using qPCR. The identified differentially expressed transcripts mRNAs were divided into three groups: Pre vs. Pro, Immature vs. Pre and Mature vs. Immature. **(A, B)** Pre vs. Pro, **(C, D)** Immature vs. Pre, **(E, F)** Mature vs. Immature. mRNA expression was quantified relative to the gapdh expression level using the comparative cycle threshold method. Columns represent means ± SEM; ****p < 0.0001, ***p < 0.001, **p < 0.01, *p < 0.05.

We validated our RNA-seq data through qPCR performed using a new cohort of animals. For each expression pattern (Pre vs. Pro, Immature vs. Pre and Mature vs. Immature), we selected 10 circRNAs, 10 miRNAs, and 10 mRNAs genes (5 upregulated genes, 5 downregulated genes). The expression analyses performed on the selected genes yielded results that were superimposable with the results obtained using RNA-seq (**Figures 5**–**7**).

### Construction of circRNA-Associated ceRNA Networks at Distinct B-Cell Developmental Stages

**Figure 8:**
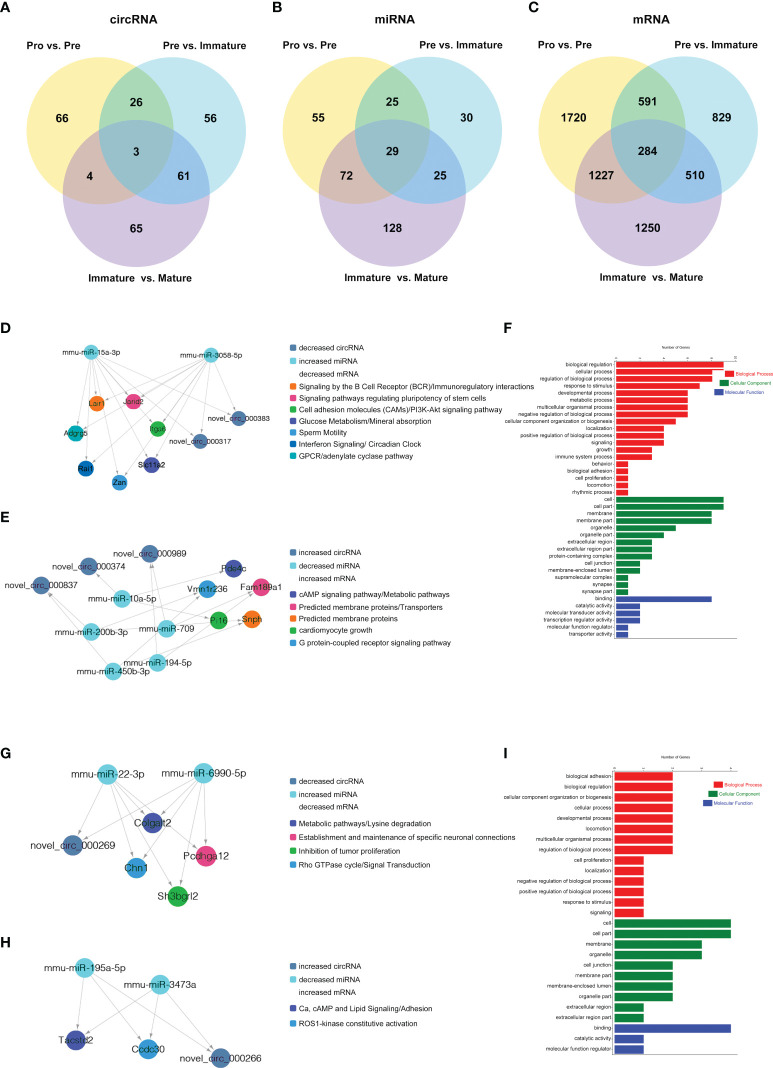
CircRNA-associated ceRNA networks and function enrichment analyses at the transitions of pro-B into the pre-B cell stage and the pre-B to immature B cell stage. ceRNA networks were constructed based on circRNA-miRNA and miRNA-mRNA interactions **(A–C)** Grouping. **(D–I)** The networks and function enrichment analyses in the pro-B to pre-B cell transitional stage (Pre vs. Pro group) **(D–F)** and the pre-B to immature B cell transitional stage (Immature vs. Pre group) **(G–I)**. **(D, G)** The ceRNA interaction of decreased circRNAs-increased miRNAs-decreased mRNAs, **(E, H)** The ceRNA interaction of increased circRNAs-decreased miRNAs-increased mRNAs. **(F, I)** GO enrichment analysis of the related genes in the ceRNA networks includes three aspects: Biological Process, Cellular Component, and Molecular Function.

The competing endogenous RNAs (ceRNAs) hypothesis indicates that ceRNA can regulate the expression of downstream genes by competing with miRNA for the common miRNA response elements (MREs). RNA-Seq data were used to construct ceRNA networks of B-cell lineage in mouse bone marrow. Here the differentially expressed transcripts (circRNAs, miRNAs, and mRNAs) were divided into three groups: (1) Pre/Pro (+) other (-): differential expression in Pre vs. Pro group but not in other groups (**Figure 8A**); (2) Immature/Pre (+) other (-): differential expression in Immature vs. Pre group but not in other groups (**Figure 8B**); (3) Mature/Immature (+) other (-): differential expression in Mature vs. Immature group but not in other groups (**Figure 8C**).

**Figure 9:**
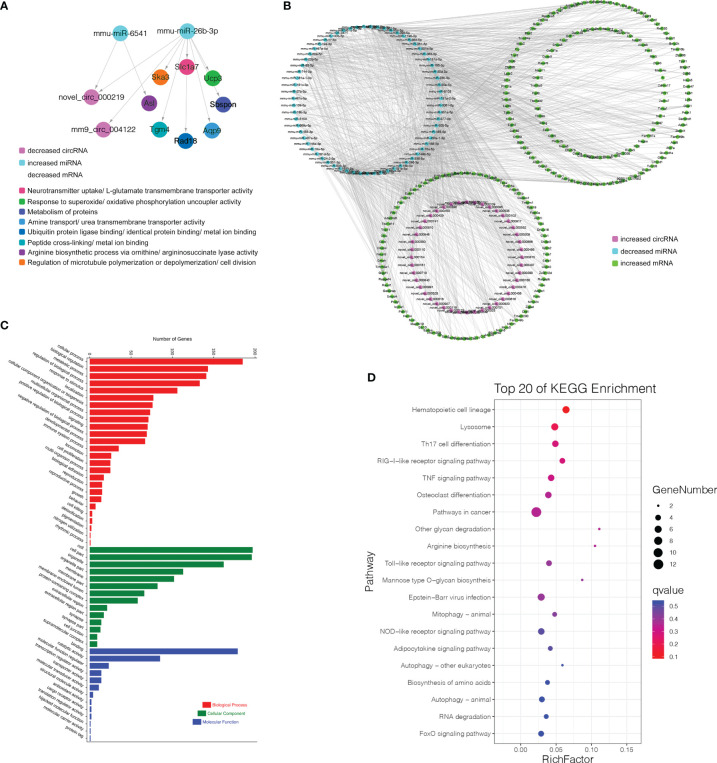
CircRNA-associated ceRNA networks and function enrichment analyses at the immature stage into mature B Cells. **(A)** The ceRNA interaction of decreased circRNAs-increased miRNAs-decreased mRNAs. **(B)** The ceRNA interaction of increased circRNAs-decreased miRNAs-increased mRNAs. **(C)** GO enrichment analysis of the related genes in the ceRNA networks. **(D)** KEGG pathways analysis of the related genes in the ceRNA networks. The Y-axis represents the pathway and the X-axis label represents the rich factor. The color and size of the bubble represent enrichment significance and the amount of differentially expressed genes enriched in the pathway, respectively.

Finally, 5 circRNAs, 7 miRNAs and 12 mRNAs significantly dysregulated in the Pre/Pro (+) other (-) group were selected to construct ceRNA networks (**Figures 8D, E** and **Table S7**). In the Immature/Pre (+) other (-) group, 2 DEcircRNAs and 6 DEmRNAs shared common MREs binding sites of 4 DEmiRNAs in this group (**Figures 8G, H** and **Table S7**). For the Mature/Immature (+) other (-) group, a total of 46 DEcircRNAs, 69 DEmiRNAs and 231 DEmRNAs were selected to construct the ceRNA networks (**Figures 9A, B** and **Table S7**). **Figures 8D, G**, **9A** show the downregulated circRNAs, upregulated miRNAs and downregulated mRNAs, while **Figures 8E, H**, **9B** show upregulated circRNAs, downregulated miRNAs and upregulated mRNAs in the ceRNA networks. It is worth mentioning that both novel_circ_000317 and novel_circ_000383 may bind mmu-miR-3058-5p and mmu-miR-15a-3p, respectively, in the pro-B to pre-B cell transition stage, which competes against their target Lair1 (**Figure 8D**). In the immature B to mature B cell stages, novel_circ_000150 might be miRNA sponge of mmu-miR-130b-5p, mmu-miR-148a-5p, mmu-miR-18b-3p and mmu-miR-467e-5p, which competes against their target complement receptor 2 (Cr2/CD21) (**Figure 9B**). Consequently, these RNA interactions might be critical in B-cell development and differentiation.

### Functional Enrichment Analyses of ceRNA Networks Genes at Distinct B-Cell Developmental Stages

GO and KEGG pathway enrichment analysis of the genes in the above ceRNA networks were performed to investigate their potential function at distinct B-cell developmental stages. The genes in the ceRNA networks of the Pre/Pro (+) other (-) group were associated with the biological process, including glucose metabolism, G-protein-coupled receptor signaling pathway, B-cell receptor signaling pathway, cAMP signaling pathway, regulation of pluripotency in stem cells, cell growth, etc. (**Figure 8D, E**). The GO terms were also found to be significantly enriched in the Pre/Pro (+) other (-) group, which contained biological process (BP), cellular component (CC), and molecular function (MF) (**Figure 8F**). Biological regulation (GO:0065007) and cell part (GO:0044464) were the top two terms.

The genes in the ceRNA networks of the Immature/Pre (+) other (-) group were associated with biological pathways, which included metabolic, cell adhesion, specific neuronal connections, etc. (**Figures 8G, H**). The top GO terms were biological adhesion (GO:0022610), cell part (GO:0044464), and cell part (GO:0044464) (**Figure 8I**).

More genes in the ceRNA networks of the Mature/Immature (+) other (-) group were discovered than those in other groups. As shown in **Figure 9C**, the top GO terms were cellular process (GO:0009987), cell (GO:0005623), and binding (GO:0005488). Several cognition-associated terms were also shown, which included metabolic process (GO:0008152), response to stimulus (GO:0050896), catalytic activity (GO:0003824), and organelle (GO:0043226). All enriched GO terms in the Mature/Immature (+) other (-) group were listed in **Table S8**. The top 20 ceRNA gene-related KEGG pathways were represented in this group (**Figure 9D**). The significantly enriched pathways included hematopoietic cell lineage, TNF signaling pathway, RIG-I-like receptor signaling pathway, pathways in cancer, Lysosome, osteoclast differentiation, Th17 cell differentiation and so on (details in **Table S9**).

## Discussion

The development of B lymphocytes has been investigated extensively over the past ten years. Some regulatory factors were critical in this complex but flexible process (31, 32), which included the E2A-EBF-PAX5 circuit (33, 34), IRF4 (35), FOXO1, interleukin-7 (36), Ying Yang 1 (37) and so on. Recently, it was reported that ncRNAs take part in regulating lymphocytes development (38). Moreover, the expression profile of circRNAs was provided in B-cell malignancies (39). However, little has been known about the profile of circRNAs and circRNA-associated ceRNA networks during the development of B-cell lineages. Hence, FACS was used to purify four B-cell subsets from single-cell suspensions of mouse bone marrow, then RNA-seq and miRNA-seq were performed. Meanwhile, we validated our RNA-seq data through qPCR. The highly stage-specific expression patterns of circRNAs suggested their strict regulation during B-cell differentiation, consistent with previous studies about the expression patterns of lncRNAs and miRNAs in these processes (40, 41). The highly unique miRNA profile of B-lymphocytes in the germinal center revealed upregulation of hsa-miR-125b downregulated the expression of key transcription factors, such as *IRF4* and *PRDM1/BLIMP1*, which regulated B cell terminal differentiation into plasma cells or memory B cells (40). The stage-specific expression of lncRNAs in B-cell subpopulations has also been identified (25). *BCALM (AC099524.1)*, a human B lymphocyte-specific lncRNA, took part in B-cell activation and differentiation by regulating BCR-stimulated Ca^2+^ signaling transduction proteins *PLD1* and *AKAP9* (26). These studies suggested that stage-specific expression of ncRNAs might play significant roles in the development and differentiation of B lymphocytes. In addition, the highly stage-specific expressed circRNAs could serve as the potential markers, which were related to the special characteristics of distinct B-cell subpopulations.

CircRNAs derived from back-splicing with retained introns could interact with UI small nuclear ribonucleoproteins (U1snRNPs), which enhanced transcription activities by recruiting Pol II at the promoters of their parental genes (42). The KEGG pathway analysis on the parental genes of stage-specific circRNAs showed that the BCR signaling pathway was the most attractive. BCR complex is composed of membrane-bound immunoglobulin (mIg), co-receptors of Ig α and Ig β, and auxiliary signal transduction elements, which are necessary for the whole process of B-cell development and maturation (8, 9). In our study, the BCR signaling pathway was enriched in pre-B cells, consistent with the first existence of pre-BCR in pre-B cells. Although the enriched analysis showed that circRNAs might indirectly participate in the BCR signaling pathway through regulating their parental genes, the specific BCR signaling pathway involved in distinct cell stages were not exactly the same. This is also consistent with their stage-specific expression characteristics. For example, *CD79a (Ig α)* was expressed along with *μ* heavy chain as part of the pre-BCR, one of the parental genes in the BCR signaling pathway specifically enriched in pre-B cells (43). *Grb2*, another parental gene in the BCR signaling pathway enriched in immature B cells, could regulate the magnitude of BCR signaling and the immunological synapse (44). As a parental gene in the BCR signaling pathway enriched in mature B cells, *Akt* was a key regulatory factor of Foxo1 transcriptional activity in B cells (45). On the other hand, the formation of circRNAs is influenced by alternative splicing and epigenetic modification (46). Previous studies have indicated that dynamic DNA methylation and histone methylation may affect the development of B cells (47, 48). Hence, the generation of stage-specific circRNAs might be affected by other epigenetic regulations on their parental genes.

The expression patterns of several circRNAs showed highly dynamic behavior throughout B-cell differentiation in the bone marrow. It suggested that the expression of circRNAs is precisely and tightly regulated. Moreover, these circRNAs may play particular roles in several successive developmental stages. For example, 9 circRNAs were consistently upregulated during myeloid B-cell development, while 7 circRNAs were consistently downregulated in this process. The expression patterns of these circRNAs indicated their sustained and stable regulatory roles throughout the developmental progression from the pro-B cells to mature B cells. For another example, 31 circRNAs firstly reached a relatively high level in the pre-B cells, but then continuously decreased in the latter two developmental stages. Therefore, these circRNAs may be critical factors for the fate of pre-B cells.

Based on the ceRNA hypothesis, we constructed the ceRNA networks to further explore the functions of DEcircRNAs in different developmental stages of B cells. For instance, novel_circ_000317 and novel_circ_000383 were identified as sponges of mmu-miR-15a-3p and mmu-miR-3059-5p, which target *Lair1* in the pro-B to pre-B cell transitional stage. *Lair1* was an immunoglobulin superfamily inhibitory receptor differentially expressed during human B-cell differentiation, which inhibited early B cell receptor-mediated signaling and B-cell maturation (49). The *Lair1* on activated B cells decreased immunoglobulin and cytokine production in BCR signaling, which inhibited B-cell proliferation and maturation (50). It suggested that circRNA might regulate the B-cell differentiation by affecting their immunoglobulin receptor. In addition, novel_circ_000150 may function as miRNA sponge of mmu-miR-130b-5p, mmu-miR-148a-5p, mmu-miR-18b-3p and mmu-miR-467e-5p, all of which targeted complement receptor 2 (*Cr2/CD21*). After late-immature B cells had exited the mouse bone marrow, the expression level of *Cr2* was increased during B-cell maturation, which was confirmed in our data (51). Premature expression of human *Cr2* during B-cell development cause defects in B-cell ontogeny and humoral immune response in mice (52, 53). Besides, novel_circ_000701 and novel_circ_000616 were identified as sponges of mmu-miR-542-3p, which targets *IL-4Ralpha.* A previous study had indicated that *IL-4Ralpha* controlled the development of IL-4-producing B cells (54). In summary, we found that several circRNAs functioned as potential ceRNAs to regulate miRNA-targeted mRNA during B-cell differentiation and development, bringing us some novel ideas to further explore the regulatory networks in these processes.

We found that the BCR signaling pathway, metabolic pathways, Toll-like receptors signaling, cell adhesion and other pathways were significantly enriched during B-cell differentiation in mouse bone marrow through functional enrichment analyses on the ceRNA networks. Many studies have reported that the BCR signaling pathway played a crucial role in B-cell development and lineage commitment (3, 9). Moreover, it was reported that key metabolic pathways, such as increased glucose uptake and induction of glycolysis, contributed to B cells fate and behavior (55–57). Toll-like receptors (TLRs) were one of the most important families of pattern-recognition receptors. In combination with other B-cell signaling pathways, TLR signaling plays a significant role in B-cell lineage determination and negative selection (58).

In conclusion, we provided the emerging field of circRNA biology with their first lineage-specific expression profiles during B-cell development, unveiling novel features of these elusive transcripts and inferred their important roles in B cells. Because of the lineage-specific distribution of circRNAs in B cells, these molecules will be considered potential developmental checkpoints or cell-specific markers. On the other hand, the circRNA-miRNA-mRNA interaction networks were constructed during B-cell development, which provided novel ideas for further exploration of the unclear mechanism of B-cell development. Meanwhile, our study may offer valuable resources for the ncRNA biology in lymphocytes. Further studies should aim at functional characterization of these circRNAs, verification of circRNA-miRNA-mRNA interaction networks, as well as identification of potential developmental biomarkers and checkpoints.

## Data Availability Statement

All raw and processed sequencing data generated in this study have been submitted to the NCBI GEO (https://www.ncbi.nlm.nih.gov/geo/) database under accession number GEO: GSE166124.

## Ethics Statement

The animal study was reviewed and approved by Shenzhen Peking University-the Hong Kong University of Science and Technology Medical Center.

## Author Contributions

XC and WZ designed studies and revised manuscripts. JP, SH, XR, HH, and XD carried out cell sorting and animal experiments. SH, JP, and IC performed the statistical analysis and drafted manuscripts. IC and BY performed the statistical analysis and revised manuscripts. All authors contributed to the article and approved the submitted version.

## Funding

National Natural Science Foundation of China (81874249), Guangdong Basic and Applied Basic Research Foundation (2020A1515011125, 2021A1515011558), and Shenzhen Basic Research Grants (JCYJ20180223181224405, JCYJ20180507182657867).

## Conflict of Interest

The authors declare that the research was conducted in the absence of any commercial or financial relationships that could be construed as a potential conflict of interest.

## Publisher’s Note

All claims expressed in this article are solely those of the authors and do not necessarily represent those of their affiliated organizations, or those of the publisher, the editors and the reviewers. Any product that may be evaluated in this article, or claim that may be made by its manufacturer, is not guaranteed or endorsed by the publisher.

## Supplementary Material

Supplementary Figure 1 Dynamic transcriptional profiles of circRNAs at distinct differentiation stages. 12 other expression patterns of circRNAs. The y-axis represents the gene expression level normalized according to the gene expression value at the first time point, and the x-axis represents the distinct differentiation stages of B-cell. The polylines indicate expression variance trends during stage progressions. The number of circRNAs within the patterns is displayed at the top of the picture.

Supplementary Methods Methodology for RNA extraction and qualification, library preparation, sequencing, quality control, read mapping to reference genome and expression analysis.

Supplementary Table 1 Specifically expressed circRNAs in pre-B-cell, pro-B-cell, immature B-cell, and mature B-cell stages.

Supplementary Table 2 Enriched KEGG pathways of stage-specific circRNAs in four B-cell subpopulations.

Supplementary Table 3 The expression patterns of circRNAs during B-cell development.

Supplementary Table 4 Differentially expressed circRNAs during two developmental stages.

Supplementary Table 5 Differentially expressed miRNAs during two developmental stages.

Supplementary Table 6 Differentially expressed mRNAs during two developmental stages.

Supplementary Table 7 The related-RNAs of ceRNA networks in the distinct groups.

Supplementary Table 8 Enriched GO terms in the Mature/Immature (+) other (-) group.

Supplementary Table 9 Enriched KEGG pathways in the Mature/Immature (+) other (-) group.
