# Supplementary figures and images for: Whole-Transcriptome Profiling and circRNA-miRNA-mRNA Regulatory Networks in B-Cell Development
Source: Front Immunol. 2022 Mar 21;13:812924. doi: 10.3389/fimmu.2022.812924 (PMC8978327; doi:10.3389/fimmu.2022.812924)

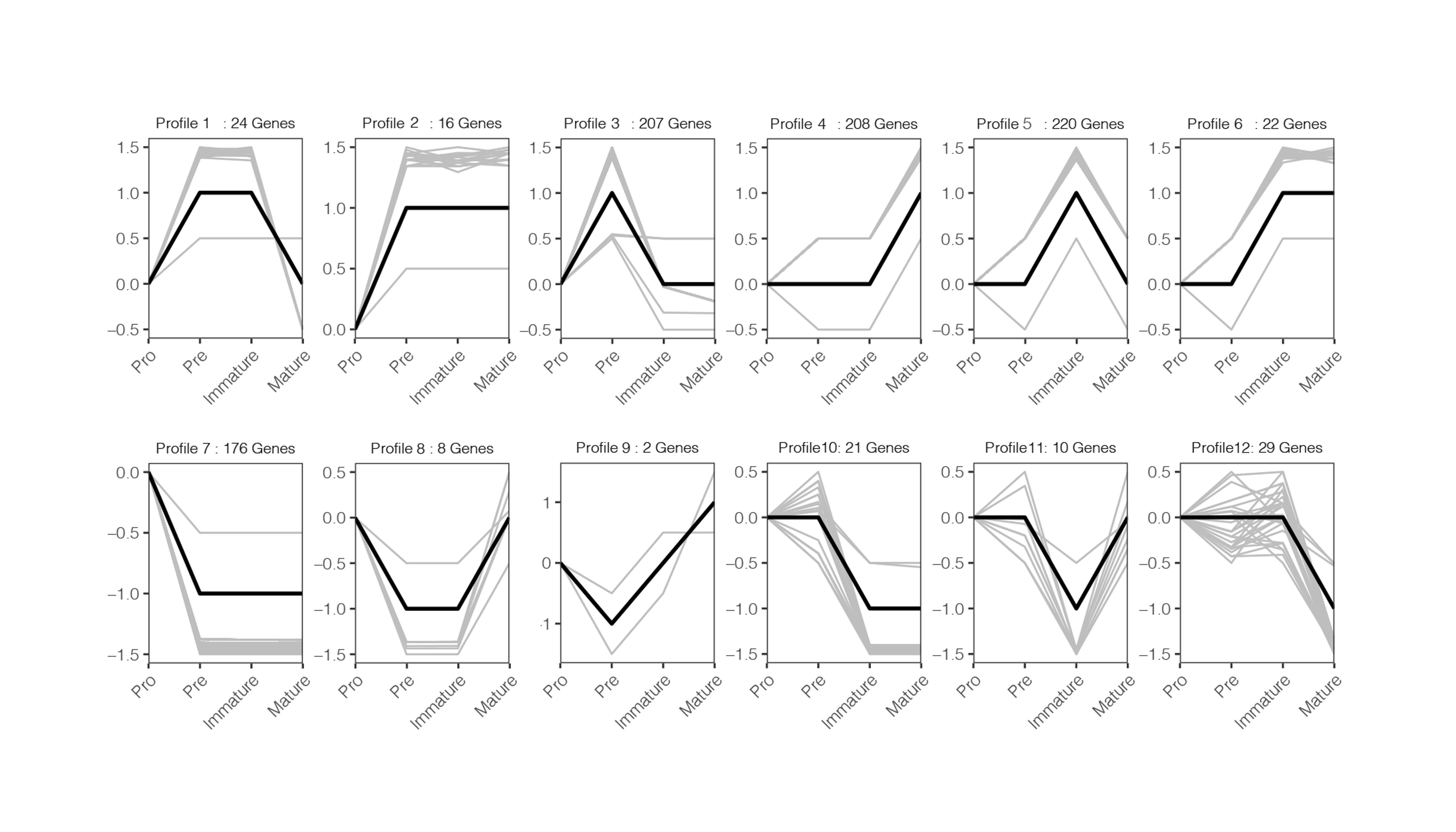

Supplement: Supplementary Figure 1 — Dynamic transcriptional profiles of circRNAs at distinct differentiation stages. 12 other expression patterns of circRNAs. The y-axis represents the gene expression level normalized according to the gene expression value at the first time point, and the x-axis represents the distinct differentiation stages of B-cell. The polylines indicate expression variance trends during stage progressions. The number of circRNAs within the patterns is displayed at the top of the picture. [file Image_1.tif]
