# Supplementary material for: Whole-Transcriptome Profiling and circRNA-miRNA-mRNA Regulatory Networks in B-Cell Development
Source: Front Immunol. 2022 Mar 21;13:812924. doi: 10.3389/fimmu.2022.812924 (PMC8978327; doi:10.3389/fimmu.2022.812924)
Supplement: Supplementary Table 1 — Specifically expressed circRNAs in pre-B-cell, pro-B-cell, immature B-cell, and mature B-cell stages. [file DataSheet_2.docx]

﻿**circRNAs**

**1 RNA extraction, library construction and sequencing**

Total RNA was extracted using Trizol reagent kit (Invitrogen, Carlsbad, CA, USA) according to the manufacturer’s protocol. RNA quality was assessed on an Agilent 2100 Bioanalyzer (Agilent Technologies, Palo Alto, CA, USA) and checked using RNase-free agarose gel electrophoresis. After total RNA was extracted, rRNAs were removed to retain mRNAs and ncRNAs. The enriched mRNAs and ncRNAs were fragmented into short fragments using fragmentation buffer and reverse transcribed into cDNA with random primers. Second-strand cDNA was synthesized by DNA polymerase I, RNase H, dNTP (dUTP instead of dTTP) and buffer. Next, the cDNA fragments were purified with QiaQuick PCR extraction kit (Qiagen), end-repaired, poly(A) added, and ligated to Illumina sequencing adapters. Then UNG (Uracil-N-Glycosylase) was used to digest the second-strand cDNA. The digested products were size selected by agarose gel electrophoresis, PCR amplified and sequenced using Illumina Novaseq6000 by Gene Denovo Biotechnology Co. (Guangzhou, China).

**2 Bioinformatics analysis**

﻿**2.1 Filtering of Clean Reads**

Reads obtained from the sequencing machines included raw reads containing adapters or low-quality bases, which would affect the following analysis. Thus, to get high-quality clean reads, reads were further filtered by fastp [1] (version 0.18.0). The parameters were as follows:

1) removing reads containing adapters;

2) removing reads containing more than 10% of unknown nucleotides(N);

3) removing low-quality reads containing more than 50% of low-quality (Q-value≤20) bases.

﻿**2.2 Alignment with Ribosome RNA (rRNA)**

﻿Different species and sample qualities would affect the efficiency of experimental ribosome RNA removal.

Thus, the short reads alignment tool Bowtie2 [2] (version 2.2.8) was used for mapping reads to the ribosome RNA (rRNA) database. The rRNA mapped reads will be removed. The remaining reads were further used in alignment and analysis.

﻿**2.3 Alignment with Reference Genome**

The rRNA removed reads of each sample were then mapped to the reference genome by HISAT2 [3] (version 2.1.1), respectively. After being aligned with the reference genome, the reads that could be mapped to the genomes were discarded, and the unmapped reads were then collected for circRNA identification.

﻿**2.4 Identification of circRNA**

20mers from both ends of the unmapped reads were extracted and aligned to the reference genome to find unique anchor positions within the splice site. Anchor reads aligned in the reversed orientation (head-to-tail) indicated circ RNA splicing and then were subjected to find_circ to identify circRNAs [4]. The anchor alignments were then extended such that the complete read aligns and the breakpoints were flanked by GU/AG splice sites. A candidate circRNA was called if it was supported by at least two unique back spliced reads at least in one sample.

﻿**2.5 circRNA statistics**

The identified circRNAs were subjected to statistical analysis of type, chromosome distribution and length distribution.

﻿**2.6 Quantification of circRNA Abundance**

To quantify circRNAs, back-spliced junction reads were scaled to RPM (Reads Per Million mapped reads), and the formula is shown as follows: **𝑅𝑃𝑀 =10^6^𝐶/𝑁**. In this formula, C is the number of back-spliced junction reads uniquely aligned to a circRNA. N is the total number of back-spliced junction reads. The RPM method can eliminate the influence of different sequencing data amounts on the calculation of circRNA expression. Therefore, the calculated expression can be directly used for comparing the differential expression among samples.

﻿**2.7 Analysis of Differentially expressed circRNAs**

To identify differentially expressed circRNAs across samples, the edgeR package [5] (version 3.12.1) (http://www.r-project.org/) was used. We identified circRNAs with a |log2 fold change (FC) | ≥1 and a P value <0.05 in comparing samples as significant differentially expressed circRNAs.

**miRNA**

**1 RNA extraction, library construction and sequencing**

After total RNA was extracted by Trizol reagent kit (Invitrogen, Carlsbad, CA, USA), the RNA molecules in a size range of 18–30nt were enriched by polyacrylamide gel electrophoresis (PAGE). Then the 3’ adapters were added and the 36-44nt RNAs were enriched. The 5’ adapters were then ligated to the RNAs as well. The ligation products were reverse transcribed by PCR amplification and the 140-160bp size PCR products were enriched to generate a cDNA library and sequenced using Illumina Novaseq6000 by Gene Denovo Biotechnology Co. (Guangzhou, China).

﻿

**2 Filtering of Clean Tags**

Reads obtained from the sequencing machines included dirty reads containing adapters or low-quality bases, which would affect the following assembly and analysis. Thus, to get clean tags, raw reads were further filtered according to the following rules:

1) Removing low quality reads containing more than one low quality (Q-value≤20) base or containing unknown nucleotides(N);

2) Removing reads without 3’adapters;

3) Removing reads containing 5’adapters;

4) Removing reads containing 3’ and 5’ adapters but no small RNA fragment between them;

5) Removing reads containing ployA in small RNA fragment;

6) Removing reads shorter than 18nt (not including adapters)

﻿

**3 Alignment and Identification of small RNA**

**3.1 Alignment with small RNA in GeneBank**

All of the clean tags were aligned with small RNAs in the GeneBank database (Release 209.0) to identify and remove rRNA, scRNA, snoRNA, snRNA and tRNA.

﻿**3.2 Alignment with small RNA in R fam**

Meanwhile, all clean tags were aligned with small RNAs in the R fam database (Release 11.0) to identify and remove rRNA, scRNA, snoRNA, snRNA and tRNA.

**3.3 Alignment with Genome (exon, intron, repeat sequences)**

All of the clean tags were also aligned with the reference genome. Those mapped to exons or introns might be fragments from mRNA degradation, so these tags were removed. The tags mapped to repeat sequences were also removed.

**3.4 Identification of microRNA (miRNA)**

**3.4.1 Identification of exist miRNA**

All of the clean tags were then searched against the miRBase database (Release 22) to identify known (Species studied) miRNAs (exist miRNAs).

**3.4.2 Identification of known miRNA**

So far, the miRNA sequences of some species were still not included in the miRBase database. The miRNAs alignment with other species was a dependable way to identify the known miRNAs for those species.

**3.4.3 Identification of novel miRNA**

All of the unannotated tags were aligned with the reference genome. The novel miRNA candidates were identified according to their genome positions and hairpin structures predicted by software mirdeep2.

﻿The default parameters of software mirdeep2 were as follows:

‑c: input file in FASTA format

‑h: parse to FASTA format

‑i: convert RNA to DNA alphabet (to map against genome)

‑j: remove all entries that have a sequence that contains letters other than a, c, g, t, u, n, A, C, G, T, U, or N.

‑k <seq>: clip 3' adapter sequence

‑l <int>: discard reads shorter than <int> nts

‑m: collapse reads

﻿‑p <genome>: map to the genome (must be indexed by bowtie-build). The genome string must be the prefix of the bowtie index. For instance, if the first indexed file is called h_sapiens_37_asm.1.ebw then the prefix is h_sapiens_37_asm.

‑q: map with one mismatch in the seed (mapping takes longer)

‑s file: print processed reads to this file

‑t file: print read mappings to this file

﻿**3.5 Small RNA annotation summary**

After tags were annotated as mentioned previously, the annotation results were determined in this priority order: rRNA etc > exist miRNA > exist miRNA edit > known miRNA > repeat > exon > novel miRNA > intron. The tags that cannot be annotated as any of the above molecules were recorded as unann.

**4 miRNA expression profiles**

Total miRNA consists of exist miRNA, known miRNA and novel miRNA; based on their expression in each sample, the miRNA expression level was calculated and normalized to transcripts per million (TPM).

﻿The formula is as follows: ﻿**TPM=Actual miRNA counts/Total counts of clean tags*10^6^**

**﻿**In addition, the expression of exist miRNA, known miRNA and novel miRNA was also analyzed individually.

﻿

**5 Differentially expressed miRNA (DE miRNA) Analysis**

miRNAs differential expression analysis was performed by edgeR software between two different samples. We identified miRNAs with a |log2 fold change (FC) | ≥1 and P-value <0.05 in comparison as significant DE miRNAs.

**mRNA**

﻿

**1 RNA Extraction, strand-specific library construction and sequencing**

Total RNA was extracted using Trizol reagent kit (Invitrogen, Carlsbad, CA, USA) according to the manufacturer’s protocol. RNA quality was assessed on an Agilent 2100 Bioanalyzer (Agilent Technologies, Palo Alto, CA, USA) and checked using RNase-free agarose gel electrophoresis. After total RNA was extracted, rRNAs were removed to retain mRNAs and ncRNAs. The enriched mRNAs and ncRNAs were fragmented into short fragments by using fragmentation buffer and reverse transcribed into cDNA with random primers. Second-strand cDNA was synthesized by DNA polymerase I, RNase H, dNTP (dUTP instead of dTTP) and buffer. Next, the cDNA fragments were purified with QiaQuick PCR extraction kit (Qiagen), end-repaired, poly(A) added, and ligated to Illumina sequencing adapters. Then UNG (Uracil-N-Glycosylase) was used to digest the second-strand cDNA. The digested products were size selected by agarose gel electrophoresis, PCR amplified and sequenced using Illumina Novaseq6000 by Gene Denovo Biotechnology Co. (Guangzhou, China).

﻿**2.1 Filtering of Clean Reads**

Reads obtained from the sequencing machines included raw reads containing adapters or low-quality bases, which would affect the following assembly and analysis. Thus, to get high-quality clean reads, reads were further filtered by fastp [1] (version 0.18.0). The parameters were as follows:

1) removing reads containing adapters;

2) removing reads containing more than 10% of unknown nucleotides (N);

3) removing low-quality reads containing more than 50% of low-quality (Q-value≤20) bases.

**2.2 Alignment with Ribosome RNA (rRNA)**

Short reads alignment tool Bowtie2 [2] (version 2.2.8) was used for mapping reads to the ribosome ﻿RNA (rRNA) database. The rRNA mapped reads were then removed. The remaining reads were further used in the assembly and analysis of the transcriptome.

﻿**2.3 Alignment with the reference genome**

An index of the reference genome was built, and paired-end clean reads were mapped to the reference genome using HISAT2 [3] (version 2.1.0) with “-rna-strandness RF” and other parameters set as a default.

**2.4 Transcripts Reconstruction**

The reconstruction of transcripts was carried out with software Stringtie [6, 7] (version 1.3.4), which together with HISAT2, allows biologists to identify new genes and new splice variants of known ones.

**2.5 Novel Transcripts Identification and Annotation**

To identify the new transcripts, all of the reconstructed transcripts were aligned to the reference genome and were divided into twelve categories by using Cuffcompare. Transcripts with one of the class codes “u, i, j, x, c, e, o” were defined as novel transcripts. We used the following parameters to identify reliable novel genes:

the length of the transcript was longer than 200bp and the exon number was more than 2 (plants more than 1).

Novel transcripts were then aligned to the Nr, KEGG, and GO database to obtain protein functional annotation

﻿**2.6 Quantification of Transcripts Abundance**

Transcripts abundances were quantified by software StringTie in a reference-based approach. FPKM (fragment per kilobase of transcript per million mapped reads) for each transcription region was calculated to quantify its expression abundance and variations using StringTie software.

The FPKM formula is shown as follows: **FPKM=10^6^C/(NL/10^3^)**

﻿Given FPKM(A) to be the expression of transcripts A, C to be the number of fragments mapped to transcripts A, N to be the total number of fragments mapped to reference genes, and L to be the number of bases on transcripts A. The FPKM method can eliminate the influence of different transcripts lengths and sequencing data amounts on the calculation of transcripts expression. Therefore, the expression of the calculated transcript can be directly used for comparing the difference of transcripts expression among samples.

﻿**2.7 Differentially expressed transcripts (DEGs) Analysis**

The differentially expressed transcripts of coding RNAs and lncRNAs were analyzed ﻿, respectively. RNAs and lncRNAs differential expression analysis was performed by edgeR [5] between two samples. P-value < 0.05 and |log2 fold change (FC) | ≥1 were set as the thresholds in the differential expression analysis.

[1] Chen, S., et al., *fastp: an ultra-fast all-in-one FASTQ preprocessor.* Bioinformatics, 2018. **34**(17): p. i884-i890.

[2] Langmead, B. and S.L. Salzberg, *Fast gapped-read alignment with Bowtie 2.* Nature Methods, 2012. **9**(4): p. 357-359.

[3] Kim, D., B. Langmead, and S.L. Salzberg, *HISAT: a fast spliced aligner with low memory requirements.* Nature Methods, 2015. **12**(4): p. 357-360.

[4] Memczak, S., et al., *Circular RNAs are a large class of animal RNAs with regulatory potency.* Nature, 2013. **495**(7441): p. 333-338.

[5] Robinson, M.D., D.J. McCarthy, and G.K. Smyth, *edgeR: a Bioconductor package for differential expression analysis of digital gene expression data.* Bioinformatics (Oxford, England), 2010. **26**(1): p. 139-140.

[6] Pertea, M., et al., *StringTie enables improved reconstruction of a transcriptome from RNA-seq reads.* Nature Biotechnology, 2015. **33**(3): p. 290-295.

[7] Pertea, M., et al., *Transcript-level expression analysis of RNA-seq experiments with HISAT, StringTie and Ballgown.* Nature Protocols, 2016. **11**(9): p. 1650-1667.
